# Intelligence gain and social cost savings attributable to environmental lead exposure reduction strategies since the year 2000 in Flanders, Belgium

**DOI:** 10.1186/s12940-019-0548-5

**Published:** 2019-12-27

**Authors:** Sylvie Remy, Ramona Hambach, Marc Van Sprundel, Caroline Teughels, Tim S. Nawrot, Jurgen Buekers, Christa Cornelis, Liesbeth Bruckers, Greet Schoeters

**Affiliations:** 1 Sustainable Health, Flemish Institute for Technological Research (VITO), Boeretang 200, 2400 Mol, Belgium; 2 Department of Epidemiology and Social Medicine, University of Antwerp, Universiteitsplein 1, 2610 Wilrijk, Belgium; 3 Department of Biomedical Sciences, University of Antwerp, Universiteitsplein 1, 2610 Wilrijk, Belgium; 4 Occupational Health Service Attentia Prevention & Protection, Keizer Karellaan 584, 1082 Brussels, Belgium; 5 Vlaams Planbureau voor Omgeving, Koning Albert II laan 20, bus 8, Brussels, Belgium; 6 Centre for Environmental Sciences, Hasselt University, Agoralaan Gebouw D, 3590 Diepenbeek, Belgium; 7 Department of Public Health & Primary Care, Leuven University, Herestraat 49 - bus 706, 3000 Leuven, Belgium; 8 I-BioStat, University of Hasselt, Agoralaan Gebouw D, 3590 Diepenbeek, Belgium; 9 University of Southern Denmark, Campusvej 55, 5230 Odense, Denmark

**Keywords:** Human biomonitoring, HBM, Lead exposure, Intelligence and economic gain, Chemical exposure prevention

## Abstract

**Background:**

Recent lead (Pb) exposure reduction strategies enabled to lower children’s blood lead levels (B-Pb) worldwide. This study reports the estimated intelligence gain and social cost savings attributable to recent exposure reduction based on reported B-Pb levels observed in adolescents sampled within the framework of the Flemish Environment and Health Studies (FLEHS, Belgium), i.e. in 2003–2004 (FLEHSI), in 2008–2009 (FLEHSII), and in 2013–2014 (FLEHSIII).

**Methods:**

Intelligence Quotient (IQ) loss per 100,000 individuals - attributable to B-Pb above 20 μg/L - was estimated based on widely accepted dose response functions between children’s B-Pb and IQ (− 1.88 IQ points for a duplication in B-Pb from 20 μg/L onwards; 95% Confidence Interval (CI): − 1.16;-2.59) and B-Pb exposure distribution parameters of FLEHS studies. The results were translated to the Flemish population of 15-year-olds. Given a 3-year time gap between subsequent sampling periods, the exposure distribution of each study was assumed 3 years prior to the study as well. Economic impact was estimated based on expected decrease in lifetime earnings (€ 19,464 per decreasing IQ point in 2018).

**Results:**

The percentage of the adolescent population exceeding a B-Pb of 20 μg/L decreased from 57% (FLEHSI) to 23% (FLEHSII), and even further to 2.5% (FLEHSIII). The estimated IQ loss per 100,000 individuals was 94,280 (95% CI: 58,427-130,138) in FLEHSI, 14,993 (95% CI: 9289-20,695) in FLEHSII, and 976 (95% CI: 604–1347) in FLEHSIII. This translates into a total loss of 378,962 (95%CI: 234,840-523,091) IQ points within the Flemish population of 15-year-olds between 2000 and 2014. Assuming that current exposure levels do not reincrease, the expected IQ loss during the subsequent period of 15 years is estimated to be maximally 10,275 (95%CI: 6363-14,182) points.

**Conclusions:**

7176 (95%CI: 4447-9905) million € of social cost savings were achieved by Pb reduction strategies in Flanders over 15 years. If current exposure levels further reduce to B-Pb below 20 μg/L for the whole population, social cost savings may increase up to 7376 (95%CI: 4571-10,181) million €. Given the relatively low lead contamination in Flanders, the global impact of ongoing reduction strategies is expected to be tremendous.

## Background

Lead (Pb) is a toxic metal that, during a lifetime, accumulates in the human body [1]. Asymptomatic health effects, such as neurotoxic effects, may occur at very low doses –below blood lead levels of 50 μg/L– and may evolve into adverse health effects if the exposure persists during a person’s lifetime or during susceptible exposure windows such as childhood seeing the development of the brain. Exposure to lead has been associated with diminished general intelligence, impaired visuomotor and cognitive ability development, and behavioral problems in young children as well as in adolescents [2–5]. It shall be emphasized that even the effects of very low environmental exposure to lead are not negligible [6, 7]. Prevention strategies have enabled a tremendous decrease in exposure in the last decades. Lead additives have been banned from gasoline since 2000 (EU directive 98/70/EG). Lead-based paint has been prohibited, although lead paint may still be found in older properties. Campaigns are ongoing to raise awareness among the general public that homes built before the 1970s are likely to have lead pipes, fixtures and solder in water supply systems. Efforts are made to replace such systems and hence reduce exposure. Furthermore, lead exposure has been associated with socio-economic status, smoking and other environmental factors. Targeted prevention strategies focusing on those factors shall be considered to further reduce exposure. Lead has been monitored extensively in European children and teenagers over the last years, though assessment of blood lead levels (B-Pb) in countries such as Belgium [8], Croatia [9], Czech Republic [10], Germany [11], Hungary [12], Kosovo [13], Poland [14, 15], Slovakia [9], Slovenia [9], and Sweden [9]. Within the framework of the Flemish Environment and Health Studies (FLEHS), B-Pb has been monitored in teenagers between 2003 and 2014 in Flanders, Belgium [8]. Based on those data, we assessed the IQ gain and cost savings that can be attributed to reductions in lead exposure over the last decade.

## Methods

### Study population

The study population has been described before [8]: 14–15-year old adolescents were sampled from the Flemish population in 2003–2004 (FLEHSI), 2008–2009 (FLEHSII), and 2013–2014 (FLEHSIII). In FLEHS I, 1679 adolescents were recruited by a randomized two stage sampling design in nine areas in Flanders with a different pollution pressure (two industrial sites, two harbors, two cities, a rural area, a zone around waste incinerators and a fruit cultivating area). In total, the area in which the participants were recruited covered 3035 km^2^ and 1.2 million inhabitants which is equal to 22% of the Flemish region and 20% of the Flemish population, respectively. In FLEHS II and III, a representative sample of the general Flemish adolescent population of respectively 210 and 208 participants was recruited by a two stage sampling design with the five districts of Flanders as primary sampling units and schools as secondary sampling units. The number of participants per province was proportional to the number of inhabitants of that province. To account for seasonal variation, recruitment was spread over one year with no recruitment of adolescents during examination periods and summer holidays (June, July, August). The studies were approved by the medical–ethical committee of the University of Antwerp (reference number A03 053, UA A08 09, and B300201316515). For all three surveys, inclusion criteria were (1) residing at least 10 years in Flanders (FLEHSII and III) or at least 5 years in one of the selected areas (FLEHSI), (2) giving written informed consent,(3) being able to fill out an extensive Dutch questionnaire, and (4) being in the 3rd year of secondary school. More detailed information on the selection and recruitment of the participants in the studies was described earlier [16–19].

### Exposure assessment

Peripheral blood samples were collected during a clinical examination in the schools. Blood lead was measured by high resolution inductively coupled plasma-mass spectrometry (HR-ICP-MS,Thermo Element II) after micro-wave acid digestion using HNO_3_ and H_2_O_2_ [8, 20].

### Blood lead level distribution

B-Pb levels reported by Schoeters et al. [8] enabled to derive the log-normal B-Pb distribution for each sampling period (FLEHSI, FLEHSII, and FLEHSIII). The data of FLEHS I were weighed to correct for unequal sample sizes in the eight geographical areas spread over Flanders. The geometric mean (GM) adolescent B-Pb in FLEHSI, FLEHSII, and FLEHSIII were respectively 22.5 μg/L (95% Confidence Interval (CI): 21.8–23.3), 14.6 μg/L (95% CI: 13.8–15.5), and 9.5 μg/L (95% CI: 9.0–9.9). The results were adjusted for age, sex, and smoking to ensure comparability between the studies. The number of participants during the first, second, and third cycle were respectively 1679, 210, and 208. The participants age ranged from 13.8 to 17.2 years of age with participants between 14.5 and 15.5 years the strongest represented (about 60%). Sex was equally distributed throughout the different studies. Smoking was reported by about 13–14% of the participants.

### Estimation of IQ loss attributable to blood lead levels above 20 μg Pb/L

To estimate the IQ loss attributable to elevated blood lead levels in the study populations we used a dose response function from the available literature that describes IQ decrement as a function of B-Pb. We focused on the study of Lanphear et al. (2005) [7] as it covered the low exposure range, it was based on international pooled analysis of 7 cohorts (total *N* = 1333), and the data had been obtained from children followed from birth or infancy until 5–10 years of age.

**Fig. 1:**
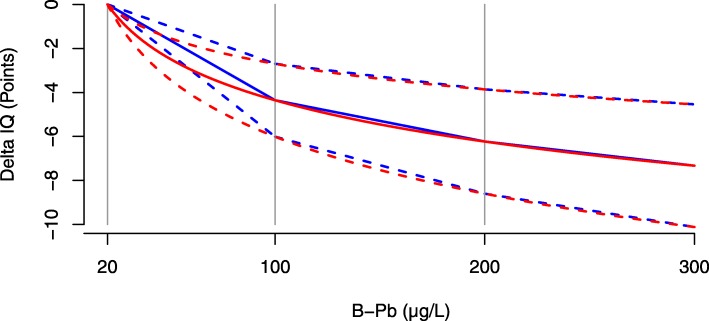
Dose response function: Inverse relationship between IQ and blood lead levels (B-Pb) for B-Pb between 20 μg/L and 300 μg/L. The red line represents the linear-log dose response relationship as estimated based on pooled international data by Lanphear et al. (2005) [7]. The blue line represents the linear-interval dose response relationship as suggested by Gould et al. (2009) [22] which assumes uniform decreases between 20 and 100 μg/L, between 100 and 200 μg/L and above 200 μg/L. The dotted lines represent the 95% confidence limits for both dose response relationships

They established a linear-log inverse relationship between IQ and B-Pb, in which a doubling in B-Pb was associated with a decrease in IQ of 1.88 points (95% CI: 1.16–2.59). The linear-log relationship implies that, for a given absolute increase in blood lead, the associated IQ loss is higher in the low level range. A statistical re-evaluation of the data used confirmed that the effect was higher in the low level range and that the conclusions were robust [21]. Based on the linear-log relationship proposed by Lanphear, Gould et al. suggested in 2009 that a uniform decrease (i.e. linear relationship) may be assumed over three ranges, i.e. for B-Pb between 20 and 100 μg/L; between 100 and 200 μg/L; and between 200 and 300 μg/L [22]. The estimated decrease in IQ points for an increase in B-Pb with 1 μg/L was equal to 0.054 (95% CI: 0.034–0.075); 0.019 (95% CI: 0.012–0.026); 0.011 (95% CI: 0.007–0.015) for blood levels between respectively 20 and 100 μg/L; 100 and 200 μg/L; and between 200 and 300 μg/L. There is little difference in the linear-log and the linear-interval dose response relationship at higher exposure levels. However, the linear-interval dose response relationship is more conservative in the lower dose range (blood lead level below 100 μg/L), as visualised in Fig. 1.

We estimated IQ loss above the threshold of 20 μg/L as dose response estimates are lacking in the lower dose region. Furthermore, 20 μg/L is considered as a relevant action level for B-Pb, as was already argued by Gilbert and Weiss in 2006 to lower the CDC action level to 20 μg/L [23]. We applied the linear-log dose response function to derive our main conclusions, the linear-interval dose response function was used as a sensitivity analysis. Based on the dose response relationships, the average IQ loss (and 95% CI) per individual within the population exceeding 20 μg/L for each sampling period was calculated by simulating 1,000,000 samples from the log-normal B-Pb distributions and taking the average. This was subsequently multiplied by the fraction of the population with B-Pb exceeding 20 μg/L and by 100,000 to estimate the IQ loss - attributable to B-Pb above 20 μg/L - per 100,000 individuals in Flanders. Although reliable dose response functions are lacking below 20 μg/L, it has been argued that there is no safe threshold for B-Pb [24]. Hence, we calculated potential additional IQ loss below 20 μg/L, by extrapolating the linear dose response function reported by Gould et al. [22] for the range between 20 μg/L and 100 μg/L.

Next, we compared the estimated IQ loss - attributable to B-Pb above 20 μg/L - between 2000 and 2014 with the estimated IQ loss that is expected between 2015 and 2029 for the Flemish population. The FLEHSI exposure distribution was fixed for the period between 2000 and 2004, the FLEHSII exposure distribution for the period between 2005 and 2009, and the FLEHSIII exposure distribution for the period between 2010 and 2014 and also for the period between 2015 and 2029. Although a further decrease in B-Pb between 2015 and 2029 may be expected, it is uncertain to what extent. Hence, the calculations for 2015–2029 may be considered as an upper limit. For each sub-period, the IQ loss and economic loss attributable to B-Pb above 20 μg/L in Flanders were calculated by taking into account the number of Flemish 15 year-olds: The average IQ loss (and 95% CI) per individual within the population exceeding 20 μg/L – calculated as described in the previous paragraph – was multiplied by the fraction of the population with B-Pb exceeding 20 μg/L and with the number of Flemish 15 year-olds within a given period. According to demographic data obtained from http://statbel.fgov.be, the number of Flemish 15 year-olds was 340,355 in 2000–2004, 364,704 in 2005–2009, and 347,761 in 2010–2014, which sums up to 1,052,820 for the period between 2000 and 2014. For the period from 2014 to 2029 we assumed that the number of 15 year-olds remains constant as the differences between the different sub-periods from 2000 to 2014 were relatively small.

### Estimation of social costs attributable to blood lead levels above 20 μg Pb/L

Social costs of IQ decrement were valued by calculating lifetime earning loss per person. We used the estimated lifetime value of 1 IQ point reported by Bellanger et al. [25] as a basis. In this publication, the authors used the life time value of 1 IQ point that was calculated for France in 2008 based on data from the US by Pichery et al. [26] (€ 17,363) and adjusted it for differences in purchasing power to derive an estimate for other countries. As such, the Belgian lifetime value of 1 IQ point was estimated at € 16,458. Based on the harmonised index of consumer prices (https://ec.europa.eu/eurostat/web/hicp/data/database) this value was adjusted for inflation, which results in an estimated lifetime value of 1 IQ point of € 19,464 for Belgium in 2018.

It shall be noted that this estimate is mainly based on studies carried out in the United States [22, 27],so we assumed that differences in lifetime incomes are the same in Europe which is not necessarily true. Adjustment for differences in purchasing power parity has been included to take this issue partially into account. As we focused on lifetime earnings only, our estimate is probably an underestimate of the total benefits of Pb control. We did not consider direct medical costs linked to treatment or interventions for children with neurodevelopmental disorders, costs related to special education or additional years of schooling for children as a consequence of these disorders [25]. Furthermore, our estimate is an underestimate of total costs attributable to Pb exposure, as we did not consider e.g. cardiovascular effects.

## Results

**Fig. 2:**
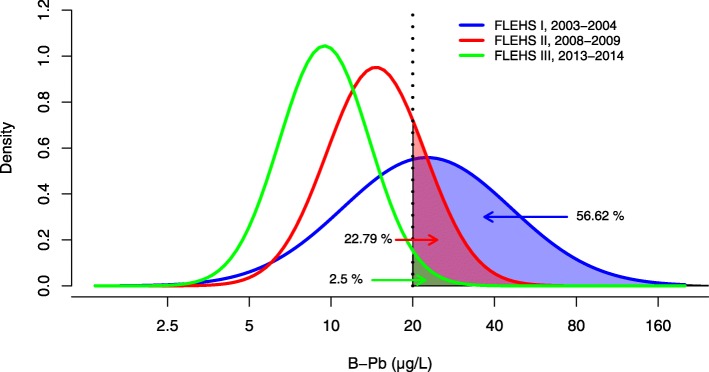
Blood lead levels in the Flemish Environment and Health Studies: Probability density function of blood lead levels (B-Pb) in adolescents sampled during the Flemish Environment and Health Studies (FLEHS). The shaded area demonstrates the percentage of the population with B-Pb above 20 μg/L (vertical dotted line) which is annotated by the arrows

The geometric mean adolescent blood Pb levels in FLEHSI, FLEHSII, and FLEHSIII were respectively 22.5 μg/L (95% CI: 21.8–23.3), 14.6 μg/L (95% CI: 13.8–15.5), and 09.5 μg/L (95% CI: 9.0–9.9) [8]. The log-normal distributions are visualized in Fig. 2, and enable to derive the fraction of the population exceeding 20 μg/L, i.e. 57% in FLEHSI; 23% in FLEHSII; and 2.5% in FLEHSIII. In FLEHSI, B-Pb levels exceeded 100 μg/L in 1.93% of the population, while this fraction was less than 0.001% in FLEHSII and FLEHSIII.

For each of the sampling periods, the estimated IQ loss attributable to blood lead levels above 20 μg Pb/L is summarized by Table 1. Based on the linear-log dose response function [7], IQ loss per 100,000 individuals was estimated at 94,280 IQ points (95% CI: 58,427-130,138) for FLEHSI; 14,993 IQ points (95% CI: 9289-20,698) for FLEHSII; and 976 IQ points (95% CI: 604–1347) for FLEHSIII. This translates into associated social costs that were nearly 100 fold reduced between 1st and 3rd cycle, i.e. from € 1835 million to € 19 million per 100,000 individuals. When using the linear-interval dose response function as sensitivity analysis, the estimates for the 3 cycles were respectively 32, 50, and 54% lower. Extrapolating the linear dose response function below 20 μg/L, an additional amount of respectively 29,917; 54,597; and 52,219 IQ points would be lost per 100,000 individuals over the 1st, 2nd, and 3rd cycle.

**Table 1:** Estimated IQ and economic loss attributable to blood lead levels above 20 μg/L: Estimates per 100,000 individuals in FLEHSI, FLEHSII, and FLEHSIII as assessed by the linear-log and the linear-interval dose response relationship

Dose Response Relationship	Study – Sampling period	Proportion population B-Pb > = 20 μg/L (%)	Average IQ-loss per individual (points) with B-Pb ≥ 20 μg/L	IQ-loss per 100,000 individuals (points) attributable to B-Pb ≧ 20 μg/L^a^	Cost per 100,000 individuals attributable to B-Pb ≧ 20 μg/L (10^6^ EUR)^a,b^
Linear-log	FLEHSI 2003–2004	56.62	1.67 (95% CI:1.03–2.3)	94,280 (95% CI: 58,427-130,138)	1835 (95% CI: 1137-2533)
	FLEHSII 2008–2009	22.79	0.66 (95% CI:0.41–0.91)	14,993 (95% CI: 9289-20,695)	292 (95% CI: 181–403)
	FLEHSIII 2013–2014	2.50	0.39 (95% CI:0.24–0.54)	976 (95% CI: 604–1347)	19 (95% CI: 12–26)
Sensitivity analysis: Linear-interval	FLEHSI 2003–2004	56.62	1.14 (95% CI:0.71–1.57)	64,421 (95% CI: 39,923-88,913)	1254 (95% CI: 777–1731)
	FLEHSII 2008–2009	22.79	0.33 (95% CI:0.2–0.46)	7539 (95% CI: 4672–10,404)	147 (95% CI: 91–203)
	FLEHSIII 2013–2014	2.50	0.18 (95% CI:0.11–0.25)	451 (95% CI: 279–622)	9 (95% CI: 5–12)

*Abbreviations*: *B-Pb* Blood lead level, *CI* Confidence Interval

^a^Taking into account the fraction of the population having B-Pb ≥ 20 μg Pb/L

^b^Based on lifetime value of 1 IQ point, i.e. € 19,464 (euro 2018)

In Table 2, the estimated IQ loss - attributable to B-Pb above 20 μg/L - between 2000 and 2014 is compared with the estimated IQ loss that is expected between 2015 and 2029 for the Flemish population. Based on the linear-log dose response relationship we calculated that the estimated average loss attributable to B-Pb between 2000 and 2014 was 378,962 IQ points (95%CI: 234,840-523,091) while a loss of 10,275 IQ points (95%CI: 6363-14,182) is expected between 2015 and 2029. For this specific population, this translates to an economic loss of € 7376 million (95%CI: 4571-10,181) between 2000 and 2014 and of € 200 million (95%CI: 124–276) from 2015 to 2029. In other words, when considering the linear-log dose relationship, the health and economic benefit attributable to Pb reduction strategies is estimated at respectively 368,687 (378,962-10,275 = 368,687) IQ points (95%CI: 228,476-508,909) and € 7176 (7376–200 = 7176) million (95%CI: 4447-9905).

**Table 2:** Estimated IQ and economic cost over a 15-year period in Flanders, Belgium: Comparison of IQ decrement and economic costs attributable to blood lead levels above 20 μg/L within the Flemish population of 15 year-olds between 2000 and 2014, and from 2015 to 2029

Dose response relationship	Time span	Number of 15 year-olds in study period	Proportion population B-Pb > = 20 μg/L (%)	Number of 15 year-olds with B-Pb > = 20 μg/L	Average IQ-loss (points) per individual with B-Pb > = 20 μg/L	IQ-loss (points) attributable to B-Pb > = 20 μg/L^a^	Cost attributable to B-Pb > = 20 μg/L (milj. EUR)^a,d^
Linear-log	FLEHSI (2000–2004)	340,355	56.62	192,717	1.67 (95% CI:1.03–2.3)	320,888 (95%CI: 198,859-442,931)	6246 (95%CI: 3873-8621)
	FLEHSII (2005–2009)	364,704	22.79	83,129	0.66 (95% CI:0.41–0.91)	54,680 (95%CI: 33,878-75,475)	1064 (95%CI: 659–1469)
	FLEHSIII (2010–2014)	347,761	2.50	8689	0.39 (95% CI:0.24–0.54)	3394 (95%CI: 2102-4684)	66 (95%CI: 41–91)
	*2000–2014*	*1,052,820*				*378,962 (95%CI: 234,840-523,091)*	*7376 (95%CI: 4571-10,181)*
	*2015–2029*	*1,052,820*^*c*^	2.50^*c*^	26305^b^	0.39 (95% CI:0.24–0.54)^*c*^	*10,275 (95%CI: 6363–14,182)*	*200 (95%CI: 124–276)*
Sensitivity analysis: Linear-interval	FLEHSI (2000–2004)	340,355	56.62	192,717	1.14 (95% CI:0.71–1.57)	219,260 (95%CI: 135,881-302,619)	4268 (95%CI: 2645-5890)
	FLEHSII (2005–2009)	364,704	22.79	83,129	0.33 (95% CI:0.2–0.46)	27,495 (95%CI: 17,041-37,944)	535 (95%CI: 332–739)
	FLEHSIII (2010–2014)	347,761	2.50	8689	0.18 (95% CI:0.11–0.25)	1569 (95%CI: 972–2164)	31 (95%CI: 19–42)
	*2000–2014*	*1,052,820*				*248,323 (95%CI: 153,893-342,726)*	*4833 (95%CI: 2995-6671)*
	*2015–2029*	*1,052,820*^*c*^	2.50^*c*^	26,305^b^	0.18 (95% CI:0.11–0.25)^*c*^	*4750 (95%CI: 2942-6550)*	*92 (95%CI: 57–127)*

*Abbreviations*: *B-Pb* Blood lead level, *CI* Confidence Interval

^a^Taking into account the fraction of the population having B-Pb ≥ 20 μg Pb/L

^b^estimated based on percentage of adolescents exceeding 20 μg Pb/L in FLEHSIII (2.50%) and total number of 15 year-olds in the period 2000–2014 (1052820): 2.5% × 1,052,820 = 26,305

^c^set equal to values FLEHSIII

^d^Based on lifetime value of 1 IQ point, i.e. € 19,464 (euro 2018)

As a sensitivity analysis, the linear-interval dose response function [22] was applied. The results are included in Tables 1 and 2 and may be interpreted in similar manner as described for the linear-log dose response function. The estimated health and economic benefit calculated is about 30% smaller as compared to the estimated benefit calculated by applying the linear-log dose response function.

## Discussion

This study provides the estimated IQ gain and cost savings that can be attributed to reductions in lead exposure over the last decade in Flanders, a West-European region, based on adolescent B-Pb levels that were measured in 2003–2014 that showed a clear decrease in B-Pb over time [8]. The cost attributable to B-Pb exposure above 20 μg/L decreased by nearly 100 fold over 10 years. Simulating the IQ decrement for the period between 2000 and 2014 and comparing it with the period from 2015 to 2029 the health and economic benefit in Flanders attributable to Pb reduction strategies has been estimated at respectively 368,687 IQ points (95%CI: 228,476-508,909) and € 7176 million (95%CI: 4447-9905). The economic estimates are strongly influenced by the shape of the dose response curve. The linear-interval curve provides about one third lower estimates as compared to the log-linear curve as the decrease in IQ per μg/L is higher in the very low level range close to the cut-off of 20 μg/L. It is important to note that the current (FLEHSIII) levels were assumed to apply for the period between 2015 and 2029. As we may assume that B-Pb will further decrease in time, the impact of Pb exposure control will be even larger. The dose response function that was used is valid from 20 μg/L onwards. By calculating the IQ decrement attributable to B-Pb above 20 μg/L we avoided to extrapolate the curve below this level. Between FLEHSI and FLEHSIII, the fraction of the population with B-Pb above 20 μg/L decreased from 57 to 2.5%. This implies that the current IQ loss attributable to B-Pb above 20 μg/L is relatively small. However, as no safe level for lead exposure in children has been identified [28], we may assume that every effort to further reduce B-Pb is beneficial.

Our study underestimates the total costs of Pb exposure, as only the value of IQ decrement was considered based on lifetime earnings.

A limitation of the study is that our result are dependent on a dose response relationship that has been established for B-Pb in children [7]. It has been well described that absorption of lead via the gastrointestinal system is higher in children as compared to older age groups [1]. Absorption of ingested lead can be up to five times greater in children than in adults and even greater when intakes of dietary minerals are deficient. B-Pb levels at 6 years of age are more strongly associated with cognitive and behavioral development than is B-Pb measured in early childhood [29]. Although the age of 6 is unlikely to represent the most sensitive age, lead concentrations may be more stable and hence more predictive [30]. Is it thus justified to use B-Pb distribution in adolescents to assess population impact on IQ? In fact, the above does not necessarily imply large differences in B-Pb distribution between children and adults. Fewtrell et al. analyzed the available literature of B-Pb levels in children and adults up to the end of 2000 in urban areas while excluding occupational exposures and studies of “hotspots” [31]. Regional urban mean B-Pb varied widely across the globe, however the differences between adults and children were small. B-Pb levels were reported for different WHO regions. For WHO region A – of which Belgium is part of – data from Denmark [32], France [33], Germany [34], Greece [35], Israel [36], and Sweden [37] were used. The geometric mean B-Pb in children was 35 μg/L, while 37 μg/L in adults. For both age groups the percentage of individuals with B-Pb above 50 μg/L was about 25%, with B-Pb between 100 and 200 μg/L was about 5% and only a small fraction exceeded 200 μg/L. In 2013, Health Canada published detailed B-Pb statistics over different age groups for Canada (2007–2009, Canadian Health Measures Survey (CHMS)) and the US (2007–2009, NHANES) [24]. For Canada, the geometric mean B-Pb was 11% lower in teenagers (12–19 years, *N* = 949) as compared to children (6–11 years, *N* = 910), i.e. 9.0 μg/L in children and 8.0 μg/L in teenagers. The 95th percentile was 19.5 μg/L in children and 16.4 μg/L in teenagers. For the US, the difference in the geometric mean and 95th percentile between children (*N* = 1011) and teenagers (*N* = 1074) was slightly higher, i.e. 19% (9.88 in children compared to 8.00 μg/L in teenagers) and 24% (25 μg/L in children compared to 19 μg/L in teenagers) respectively. These findings indicate that our results based on B-Pb in adolescents of 14–15 years old likely underestimate the actual impact of lead on IQ in the population. Since the difference between children and adolescents is not only explained by higher absorption upon oral exposure, high precision models to estimate B-Pb in children from B-Pb in adolescents are currently lacking. Differences in B-Pb in children and adolescents may be introduced e.g. by exposure to environmental tobacco smoke which has been studied by Mannino et al. in a sample of 5592 U.S. children and adolescents, aged 4–16 years, who participated in the Third National Health and Nutrition Examination Survey (NHANES) (1988–1994) [38]. The geometric mean B-Pb was 26 μg/L in 4–6 year-olds, 21 μg/L in 7–11 year-olds, and 16 μg/L in 12–16 year-olds. Although the difference in B-Pb between children and adolescents was small when cotinine was low, average B-Pb was about 75% higher in 14–15 year-olds as compared to 6 year-olds when cotinine levels where high. A critical analysis of the results showed that both cotinine and B-Pb were strongly related to socio-economic status (SES) [30]. Low SES parents may smoke more cigarettes, but also, the children are more likely exposed to different sources of lead (e.g. lead containing dust related to different housing environments). As high precision models to estimate B-Pb in children from B-Pb in adolescents are currently not available, the difference in B-Pb between adolescents and children has not been taken into account in our calculations. However, if we assume overall higher B-Pb in children, the results of our study per sampling cycle are likely underestimates. But, as we may assume that each of the exposure distributions would shift equally to the right, the impact that has been established over the years by decreasing B-Pb levels remains considerably large.

## Conclusion

This study estimated intelligence gain and social cost savings attributable to recent exposure reduction based on B-Pb levels observed in adolescents sampled between 2003 and 2014 within the framework of the Flemish Environment and Health Studies (FLEHS, Belgium). Our findings highlight that efforts that reduced Pb exposure result in a high health and economic benefit. We demonstrate that 7176 (95%CI: 4447-9905) million € of social cost savings were achieved by Pb reduction strategies in Flanders over 15 years. The results are relevant for other countries of the Western EU region as well. The approach can be used to estimate the impacts of many environmental policies.

## Data Availability

This manuscript is based on published human biomonitoring data (Schoeters G, Govarts E, Bruckers L, Den Hond E, Nelen V, De Henauw S, et al. Three cycles of human biomonitoring in Flanders − Time trends observed in the Flemish Environment and Health Study. Int J Hyg Environ Health. 2017). The data that support the findings of this study are available from the Flemish Center of Expertise on Environment and Health but restrictions apply to the availability of these data. Data are however available from the authors upon reasonable request and with permission of FLEHS supervisory committee, and while respecting the general data protection regulation (GDPR, Regulation (EU) 2016/679).
